# Ethical, legal, and social issues in the Earth BioGenome Project

**DOI:** 10.1073/pnas.2115859119

**Published:** 2022-01-18

**Authors:** Jacob S. Sherkow, Katharine B. Barker, Irus Braverman, Robert Cook-Deegan, Richard Durbin, Carla L. Easter, Melissa M. Goldstein, Maui Hudson, W. John Kress, Harris A. Lewin, Debra J. H. Mathews, Catherine McCarthy, Ann M. McCartney, Manuela da Silva, Andrew W. Torrance, Henry T. Greely

**Affiliations:** ^a^College of Law, University of Illinois at Urbana–Champaign, Champaign, IL 61820;; ^b^Carl R. Woese Institute for Genomic Biology, University of Illinois at Urbana–Champaign, Urbana, IL 61801;; ^c^Center for Advanced Studies in Biomedical Innovation Law, University of Copenhagen Faculty of Law DK-2300 Copenhagen S, Denmark;; ^d^Global Genome Initiative and Global Genome Biodiversity Network, National Museum of Natural History, Smithsonian Institution, Washington, DC 20560;; ^e^University at Buffalo School of Law, The State University of New York at Buffalo, Buffalo, NY 14260;; ^f^School for the Future of Innovation in Society and Consortium for Science, Policy & Outcomes, College of Global Futures, Arizona State University, Washington, DC 20006;; ^g^Department of Genetics, University of Cambridge, Cambridge CB2 3EH, United Kingdom;; ^h^Wellcome Sanger Institute, Hinxton CB10 1SA, United Kingdom;; ^i^Education and Community Involvement Branch, National Human Genome Research Institute, Bethesda, MD 20892;; ^j^Department of Health Policy and Management, Milken Institute School of Public Health, George Washington University, Washington, DC 20052;; ^k^Te Kotahi Research Institute, University of Waikato, Hamilton 3216, New Zealand;; ^l^Genomics Aotearoa, University of Otago, Dunedin 9016, New Zealand;; ^m^The Arnold Arboretum, Harvard University, Jamaica Plain, MA 02130;; ^n^Department of Botany, National Museum of Natural History, Smithsonian Institution, Washington, DC 20560;; ^o^Department of Evolution and Ecology, College of Biological Sciences, University of California, Davis, CA 95616;; ^p^Department of Population Health and Reproduction, University of California, Davis, CA 95616;; ^q^Berman Institute of Bioethics, Johns Hopkins University, Baltimore, MD 21205;; ^r^Department of Genetic Medicine, Johns Hopkins University School of Medicine, Baltimore, MD 21205;; ^s^Genome Informatics Section, National Human Genomics Research Institute, Bethesda, MD 20892;; ^t^Fiocruz Covid-19 Biobank, Fundação Oswaldo Cruz, Rio de Janeiro 21041-361, Brazil;; ^u^School of Law, University of Kansas, Lawrence, KS 66045;; ^v^Center for Law and the Biosciences, Stanford Law School, Stanford University, Stanford, CA 94305;; ^w^Department of Genetics, School of Medicine, Stanford University, Stanford, CA 94305

**Keywords:** genomics, ELSI, EBP, ethics, biodiversity

## Abstract

The Earth BioGenome Project (EBP) is an audacious endeavor to obtain whole-genome sequences of representatives from all eukaryotic species on Earth. In addition to the project’s technical and organizational challenges, it also faces complicated ethical, legal, and social issues. This paper, from members of the EBP’s Ethical, Legal, and Social Issues (ELSI) Committee, catalogs these ELSI concerns arising from EBP. These include legal issues, such as sample collection and permitting; the applicability of international treaties, such as the Convention on Biological Diversity and the Nagoya Protocol; intellectual property; sample accessioning; and biosecurity and ethical issues, such as sampling from the territories of Indigenous peoples and local communities, the protection of endangered species, and cross-border collections, among several others. We also comment on the intersection of digital sequence information and data rights. More broadly, this list of ethical, legal, and social issues for large-scale genomic sequencing projects may be useful in the consideration of ethical frameworks for future projects. While we do not—and cannot—provide simple, overarching solutions for all the issues raised here, we conclude our perspective by beginning to chart a path forward for EBP’s work.

The Earth BioGenome Project (EBP) is an audacious endeavor, an attempt to obtain whole-genome sequences from specimens of every eukaryotic species on Earth—land, sea, sky, or underground. We know of about 2 million such species ranging in size from the blue whale to a single-cell plankton in the class Mamiellophyceae; it is estimated that about another 7.5 million currently unknown eukaryotic species exist (1). The knowledge generated by EBP may “lead to new food sources, revolutionary bio-inspired materials, and innovations to treat human, animal, and plant diseases” (2). Also, “[i]f successful, the EBP will completely transform our scientific understanding of life on earth and provide new resources to cope with the rapid loss of biodiversity and habitat changes that are primarily due to human activities and climate change” (2).

The scientific and technical problems of finding, sampling, sequencing, databasing, and analyzing these eukaryotic genomes are enormous, but so too are the ethical, legal, and social challenges associated with the project. This perspective highlights and categorizes many of the ethical, legal, and social issues currently confronting EBP and suggests a path forward. At the same time, we recognize that the problems inherent in the complexity of interests in a project like EBP are myriad, that solutions to some of these issues may be controversial or currently unavailable, and that resolving disputes over individual sequencing projects will likely require further input, not only of EBP and its members but also the broader public as well. It is nonetheless our belief that these problems can be managed well enough to enable EBP to proceed—and to succeed—equitably and fairly for all of humanity and the biosphere.

## Legal Issues

EBP’s goal of sequencing representatives from all extant Eukarya raises a number of significant international and national legal challenges. These concern basic legal obligations on the part of researchers, such as proper sample collection and permitting, but also more complex requirements, such as the Nagoya Protocol’s requirements regarding access and benefit sharing (ABS) for the utilization of genetic resources. Beyond these obligations, EBP and its member projects face difficult questions pertaining to rights and responsibilities regarding intellectual property (IP), sample collecting practices, accessioning rules for collected samples, and biosafety and national security restrictions.

### Sample Collection and Permitting.

Sequencing a genome often requires a tissue sample from the species, and most countries have regulations governing the collection of biological samples for research. EBP’s work, by its nature, is international in scope; a great number of species are endemic to only a single country or very few (3). This means that EBP researchers, at least today, are frequently tasked with collecting samples in one jurisdiction and preparing and sequencing them in another. As discussed later in this paper, fostering the sequencing of species in the country in which they are found is a future project goal.

Many countries have biological permitting restrictions for engaging in species sample collection, some of which are the consequence of international treaties, while others are entirely domestic in nature. The Convention on International Trade in Endangered Species of Wild Fauna and Flora (CITES) is perhaps the best-known of such international treaties in this regard and regulates the import, export, and reexport of International Union for Conservation of Nature (IUCN)-listed endangered species and derived materials without prior permitting from their respective source countries (4). Beyond CITES, a number of other legal frameworks operate similarly, including the Migratory Bird Treaty Act (implementing separate conventions among Canada, Mexico, Japan, and Russia) (5), the Marine Mammal Protection Act (6), and the African Elephant Conservation Act (7).

Supranational jurisdictions, such as the European Union, have a host of similar limitations among their respective member nations (8). In addition, biological samples sourced from Antarctica, specifically, are subject to governance under the Antarctic Treaty System, which encompasses not only the Antarctic Treaty, which came into force in 1962 and now has 54 members, but also over 200 separate requirements, including those in the Protocol on Environmental Protection to the Antarctic Treaty (9). Marine samples have yet further sampling and permitting restrictions, governed in many instances by the United Nations Convention on the Law of the Sea (UNCLOS) (10) or, in the special case of cetaceans, the International Convention for the Regulation of Whaling (ICRW) (11).

Some jurisdictions, meanwhile, have purely domestic permitting requirements for species of significant national interest, such as the United States Bald and Golden Eagle Protection Act (12), and yet others may institute special national permitting processes for foreign researchers regardless of the particular species to be collected (13). In addition, some permitting processes may include requirements pertaining to vouchering—requiring a third party to maintain an archetypal specimen in an accessible collection (14).

Assessing compliance with this web of legal obligations is complex, but necessary, and EBP researchers will need to take a systematized, species-by-species, sample-by-sample, and jurisdiction-by-jurisdiction approach to ensure compliance with these laws. The costs, in terms of researcher time and effort, are likely to be nontrivial. Nonetheless, many of the protections instituted in the above laws were put in place precisely to avoid the exploitation of biological resources that is currently contributing to the global decimation of biodiversity. Others, meanwhile, are geared to share the benefits of biodiversity as a solution to extractive biocolonialism. A principal goal of EBP is to halt, if not reverse, the global decline in biodiversity; circumventing restrictions on sample collection, aside from being illegal, may be counterproductive in the context of creating benefits for society and human welfare.

### The Convention on Biological Diversity and the Nagoya Protocol.

The Convention on Biological Diversity (CBD), first signed in 1992, seeks to “conserve and sustainably use biological diversity for the benefit of present and future generations” (15) by creating a biodiversity conservation framework that binds its 196 member countries—more members than currently constitute the United Nations (16). The CBD is not self-executing, however; it requires its members to enact their own domestic laws in accordance with the Convention and designate National Focal Points responsible for their implementation. National enforcement of these domestic laws is, however, inconsistent (17). In addition, the United States is a notable holdout to the CBD, with observer status only (18). This does not mean that the US researchers are not subject to the CBD; they may still be bound by national laws in place within countries working toward CBD objectives. US researchers conducting genomic research in CBD member countries, or using samples originating from CBD member countries, are subject to those countries’ implementations of the CBD (18).

Of particular salience for EBP’s member projects is a protocol agreement enacted pursuant to the CBD, the Nagoya Protocol on Access to Genetic Resources and the Fair and Equitable Sharing of Benefits Arising from Their Utilization. The Nagoya Protocol was created with the goal that “benefits arising from the utilization of genetic resources … shall be shared in a fair and equitable way” with signatory countries and “with the aim of ensuring that benefits arising from the utilization of genetic resources that are held by indigenous and local communities, in accordance with domestic legislation … are shared in a fair and equitable way with the communities concerned, based on mutually agreed terms” (19, 20). Benefit sharing may include monetary benefits, such as access fees to samples, or nonmonetary benefits, such as institutional capacity building or fostering research and development. While the Nagoya Protocol contemplates a global multilateral benefit-sharing mechanism related to digital sequence information (DSI), none has yet been implemented. Meanwhile, a number of jurisdictions have in place their own national benefit-sharing mechanisms specific to species derived from their host countries (21). Procedures to institute global ABS mechanisms under the Nagoya Protocol are underway, and EBP research programs will likely be affected by the policies that emerge from these deliberations and other international agreements. Those participating in the EBP can—and should—help inform the parties deciding the rules.

### IP.

IP is an umbrella term that describes a suite of private property rights in the objects of research. These include patents, which prevent others from copying a claimed invention, but also trade secrets, which protect economically valuable secret information, and, in some jurisdictions, databases. EBP researchers are likely to face questions regarding if they can (and whether they should) protect the objects of their research by securing them with IP. Advances in sequencing techniques, for example, are likely amenable to patent protection, and in many jurisdictions sequence data may constitute trade secrets or be subject to database protections. Also, patent protection is largely available for products, such as medicines and research tools, derived from genomic information.

Controversially, some jurisdictions allow the patenting of genomic sequences themselves. Patents applications under the European Patent Convention, for example, can be directed to genomic sequences, if they are “produced by means of a technical process” and subject to an “industrial application” (22). In the United States, by contrast, patents covering isolated genomic sequences have largely been forbidden (23). These divergent approaches to the patenting of genomic sequences across international boundaries makes globally assessing these issues a complex endeavor.

Beyond immediate questions of patent eligibility, the extent of IP has been a thoroughly controversial topic for sequencing projects for decades, including claims of extractive biocolonialism such as the patenting of indigenous medical remedies (24). Traditional knowledge databases have emerged in countries like South Africa and India to counter some of these IP strategies. In other instances, IP has appeared to stymie the objectives of larger sequencing projects (25). Resolving these concerns alongside patent incentives for downstream products can be difficult where commercial funding is involved in sequencing efforts. Squaring researchers’ rights to seek protection for their work with the need for open access to the results of sequencing projects will remain an ongoing challenge for EBP and one that will require the EBP Ethical, Legal, and Social Issues (ELSI) Committee’s further attention.

### Sample Storage and Accessioning.

Apart from issues pertaining to the legality of collection and permitting, several international agreements also impose requirements on the storage of biological samples and accessioning, i.e., where samples are held, the provenance of samples, and who has access to them. The International Treaty on Plant Genetic Resources for Food and Agriculture, for example, requires certain samples from a list of food crops to be accessible to others through a network of International Agricultural Research Centers (26). Article 9 of the CBD analogously advocates that member countries adopt accessioning measures for ex situ samples within the “country of origin of such components” (15). This means that even where collection and permitting of species have been appropriate, EBP researchers should think ahead regarding where their underlying samples will be preserved in the long term, how samples will be curated, who should have custody over such samples, and who will have access for future research.

### National Security and Bioterrorism.

In rare instances, the collection and sequencing of certain eukaryotic species may present some issues regarding national biosecurity and biosafety. *Coccidioides immitis*, the fungal, etiological agent of valley fever, has been designated a “Select Agent” by the US Centers for Disease Control and Prevention and classified as a biosafety level 3 hazard (27). The United Kingdom, similarly, classifies more than 100 multicellular Eukarya (mostly fungi and helminths) as posing the potential for harm to human health (28). Yet other eukaryotic species produce toxins that may require extra precautions for import and control (29), while other countries have no such lists at all. Because the transfer of species across borders presents control challenges for EBP researchers, it is imperative for EBP researchers to plan the export and import of any samples carefully to ensure compliance with select agent restrictions, both from the exporting country as well as to the importing country, in addition to any countries through which the sample may pass in transit.

## Ethical Issues

Some of the work conducted under EBP’s umbrella raises ethical issues concerning equity, justice, and fairness. EBP’s underlying core mission is to enhance genomic sampling across the tree of life—not just in service of producing knowledge for knowledge’s sake but also to protect and conserve a common human heritage. Upholding EBP’s mission to conserve, protect, and restore biodiversity raises difficult ethical issues when considered alongside the benefits to society and human welfare of EBP’s work. Alongside this, there are further tensions related to the rights and responsibilities to Indigenous Peoples and their jurisdictional claims to certain species, sampling endangered species, choosing sampling sites for transnational species, museum and zoo collections, animal welfare, and what to do about unethically obtained samples. We highlight some of the most salient of these issues below.

### Indigenous Peoples and Local Communities.

Many countries recognize Indigenous peoples and local communities (IPLCs) as forming part of their nations’ constituent sovereignty, such as New Zealand’s constitutional relationship with Māori iwi, the United States’ recognition of over 500 Federal Indian Tribes, and Brazil’s demarcation of Indigenous Territories as well as its recognition of IPLCs. Where countries’ laws govern such a relationship, derivation of species from the land of IPLCs should follow those laws. However, even where there is not formal recognition of an IPLC by a sovereign authority, when there is a dispute regarding the extent of such recognition or when rights of multiple IPLCs collide, the claims of those peoples and communities should be respected. Doing so may entail recognizing rights—like those articulated in the United Nations Declaration on the Rights of Indigenous Peoples—beyond those formally required by the country in which a particular species is collected (30). This may include ensuring the sort of ABS contemplated by the Nagoya Protocol, or working with additional protections to native species or restrictions on sampling and beyond. Given the long history of bioresource depletion on the land of native peoples, this is an issue of particular concern and importance for EBP and its member projects.

### Endangered Species and Project Selection.

As of this writing, there are 1,059 species (and 36 separate subspecies) indexed by CITES as being endangered of becoming extinct; another 37,420 species (and 15 subspecies) are threatened with extinction (31). In the drive to sequence all eukaryotic genomes, there is an understandable urgency—as well as real constraints on project funding and researcher time—to focus early on these species. Encouraging such work may allow some species to be cataloged prior to their becoming extinct. At the same time, endangered species are not evenly distributed across the globe, and even among endangered species there is often a preference to sequence, first, the “charismatic megafauna” (32). Sometimes this is beneficial, as others have noted, if it can be used as an umbrella species for a larger conservation project. EBP will work with its member projects to ensure that resources can be equitably deployed to the most vulnerable of species without necessarily focusing initial sequencing efforts on those species which command the most public attention.

### Cross-Border Species/Race to the Bottom.

Many species have transnational habitats; some, like the blue whale, *Balaenoptera musculus*, or the black garden ant, *Lasius niger*, are entirely worldwide. Where species do cross borders (or exist outside of clearly demarcated national borders) there are likely to be differences in jurisdictions’ treatment of research efforts, from differing standards for sample collection and permitting to competing regimes for ABS. In an effort to catalog all Eukarya, there is pragmatic appeal to sample and sequence representative species from “easier” jurisdictions, i.e., those with lesser or easier restrictions on research and sampling. However, researchers should be sensitive to overlapping claims regarding transnational species, especially where jurisdictions more amenable to simpler research restrictions have substantially smaller populations than their neighboring countries or where claims straddle fully developed nations and low-to-middle-income ones. Ideally, such concerns will wash out, in time, as more and more species are sampled and sequenced, but this is not a given.

### Zoo and Biological Collections.

Samples collected not from species’ in situ environments but from zoos, museums, botanical gardens, herbaria, or culture collections present potential ethical challenges regarding their provenance. Questions are likely to arise regarding whether the samples collected were acquired ethically and legally, and whether they can be appropriately “repurposed” or borrowed for sequencing efforts. While such issues are most likely to arise in the context of zoos and museums, this applies equally to samples that are collected (or transferred by third parties) from ex situ sources by purchase, donation, bequest, exchange, or unsolicited submission. This is likely to require researchers, presented with such samples, to affirmatively inquire as to their provenance and to examine any appropriate documentation of the status of the materials and limitations on their uses. Researchers are also likely to face related questions for biological samples collected prior to the enactment of the CBD and should engage in a similar analysis.

### Animal Welfare.

For a number of Eukarya, pain and distress in sampling are of particular concern. As with other activities involving such animals, researchers have an ethical (and sometimes legal) obligation to minimize animal pain and distress as much as practical (33). For many larger animals, advances in minimal sample DNA sequencing technology allow researchers to conduct sequencing without causing lasting harm, e.g., by using blood samples or other minimally invasive techniques. Reduction, refinement, and replacement, as originally set out in *The Principles of Humane Experimental Technique* (33), have become a cornerstone of ethical research for many animal species and should be an integral part of any research project to help minimize animal use and suffering and to facilitate good scientific practice (34).

### Unethically Obtained Samples.

Finally, there exist broader issues about what do to about sequencing samples later found to have been unethically obtained. When researchers are presented with information to apprise them that samples used in their sequencing projects were not obtained ethically, they should investigate why and how such samples were originally obtained. If the ethical violation concerns matters unrelated to obtaining the sample for sequencing, researchers should evaluate whether to make use of such samples and how best to acknowledge and account for any ethical lapses in their acquisition. Failing to do so is likely to encourage lawlessness for collection and permitting restrictions and efforts to circumvent Nagoya’s ABS requirements.

## Societal Concerns

While many of these ethical issues will be individualized and specific to certain sequencing projects, there are also broader societal concerns regarding EBP and its expansive efforts, including other global, large-scale sequencing projects. These include the project’s role in the emerging bioeconomy, conservation efforts, community involvement and representation, cost, sharing, and oversampling.

### The Emerging Bioeconomy.

One of the three principal goals of EBP is to create new benefits for society and human welfare. Understanding biology and evolution at a global scale will create knowledge that can hopefully be applied toward solving human illness and advancing the bioeconomy. The innovation potential of these technologies and nature’s biological assets has been previously articulated by other global initiatives, such as the Earth Bank of Codes (34). At the same time, issues of equity and benefit sharing in this emerging bioeconomy continue to be widely debated, especially in the context of the CBD, the Nagoya Protocol, and among indigenous academics (31). The work of EBP should recognize its role in generating the underlying sequence data that support this economic activity, even if that is not the primary goal of each partner.

### Alignment with Conservation Efforts.

Another goal of EBP is to support conservation. Sequencing all of Eukarya will allow researchers to better understand the range and scope of this planet’s genetic biodiversity, and—as the project expands—to identify genetic bottlenecks before they contribute to population decline (35). This premise, however, is not without detractors. Some have suggested that large-scale sequencing projects only give license to anticonservationist behaviors, a simple catalog of harm that can be explained away when inconvenient (36). Relatedly, others have suggested that large-scale sequencing efforts are relatively meaningless to conservation efforts or that genetic sequencing projects are a distraction from doing meaningful conservation work (37).

We believe these moral hazard concerns are unlikely to be borne out. As has been recently documented, genetic sequencing is important—and becoming increasingly important—for conservation efforts, from conservation projects, like those pertaining to genetic bottlenecks affecting the African cheetah (38), or restoring genetic diversity to the black-footed ferret (39), or reviving the American chestnut (40). EBP intends for its work to be aligned with these efforts, complementary to all species conservation projects and necessary to some.

### Community Involvement and Representation.

EBP is a global project intended to benefit all of humanity—not just its member scientists. Where the public can identify concerns about individual sequencing projects—either their scope or their implementation—EBP should take such concerns seriously. Community involvement is also important in a practical sense: as participants and partners. This may include species identification and tracking and, with appropriate guidance and training, sample collection. The public may also help support the larger project in many ways, from identifying certain EBP projects as opportunities for community science to administrative assistance and help with publicity and sharing of results. EBP should also do its best to ensure that community partnerships and participation are wide-ranging and represent not just a diversity of species but a diversity of peoples (41).

### Cost.

Large-scale scientific research programs like EBP are generally costly—with, in most cases, the public paying for much of that cost. The public would therefore be right to question why it is worthwhile to spend money on a research project with this focus and magnitude. We think the answers are plentiful: EBP is the development and catalog of Earth’s biodiversity; it is, all things considered, rather inexpensive for its aims; it is likely to contribute to the development of innovative research tools useful in future scientific endeavors; and it has the potential—perhaps more so than other scientific efforts—to foster goodwill among nations. In addition, investment in EBP will likely yield new genomic infrastructure and resources and contribute to the expansion of the (rapidly growing) bioeconomy. Beyond these benefits, we similarly anticipate that genomic data created from EBP will assist in the development of other fields, such as synthetic biology, and improve a variety of biological tools, such as CRISPR. Ultimately, “[t]he greatest legacy of the EBP will be the gift of knowledge—a complete Digital Library of Life that contains the collective biological intelligence of 3.5 billion years of evolutionary history. This knowledge will guide future discoveries for generations and may ultimately determine the survival of life on our planet” (1).

### Sharing.

The scope of EBP also raises issues pertaining to the sharing of its output, i.e., the sharing of its data, results, and publications. As a catalog of species diversity across the globe, researchers should—as best they can—encourage the sharing of their research. This includes best efforts to ensure that underlying genomic data are both publicly available and readily accessible. The particular contours of such sharing will depend, in large part, on countries’ commitments to access with respect to ABS under the Nagoya Protocol, as well as emerging discourse around Indigenous Data Sovereignty (42). However, barring the most extreme cases of ABS constituting private databases and pay-for-access data regimes, there are likely to be opportunities for researchers to share freely as much of their data as possible while still effectuating any multilateral agreements developed under the Nagoya Protocol. Much as the Bermuda Principles spurred an open-access regime to human genomic data that benefitted all of humanity, so too can data sharing—even under an ABS framework—redound to people around the world.

### Oversampling.

An additional societal issue pertains to oversampling—the harvesting of biological samples beyond that necessary to complete the task at hand. Scientists have come a long way from the days of indiscriminate collecting famous in the 19th century. Today, in most cases, a genome sequence requires only a single individual of a species to be sampled, or at most a small number of individuals. Nonetheless, and especially for endangered species or species in environments particularly sensitive to human traffic, researchers should ensure that all sampling is substantially below levels that would affect population demography or their natural environments. While sequencing projects, like EBP, cannot “take only pictures and leave only footprints,” researchers can be attuned to the impacts of their research on their samples’ populations and the surrounding environment.

## Data and DSI

Woven throughout the issues raised above are complexities regarding the generation and sharing of the sequence data, known under the CDB and Nagoya Protocol as DSI. DSI, like all other digital material, is easily shared across borders. This complicates how DSI is, and should be, considered under the Nagoya Protocol’s ABS principles, and there are conflicting views regarding unrestricted access to genomic data and the rights and interests of nations, Indigenous peoples, and local communities to control such data (43). A meeting last year between representatives from the European Union and China summarized the potential conflict concerning DSI and Nagoya’s DSI requirements this way: “Open data [are] a key component of the smooth functioning of science globally. However, open access may restrict options to address benefit sharing and the challenge is to generate a different approach that maintains the efficiencies of the current model in delivering societal monetary and non-monetary benefits arising from activities within the current system” (44).

Assessing whether this conflict is real or hypothetical lies in the details of any DSI sharing regime. Much of the difficulty lies in narrowly conceiving of the benefits contemplated as primarily arising from a “payment for data” regime, even while there are greater opportunities for collaboration around other value-generating activities. At the same time, there are models where open data have produced monetary rewards for its generators, such as providing data hosting, developing analysis tools, or selling derivative products from such data. One relevant example might be the establishment and support of local sequencing capacity within source nations currently deprived of it and furthering training in the area. The COVID-19 pandemic has demonstrated the need to expand sequencing capacity globally. Researchers from sequencing-capacity-rich nations whose sequencing efforts will primarily focus on source nations without such resources should commit to generating solutions for this gap in sequencing capacity. Depending on how they are deployed, open data and a call for benefit sharing may not be in conflict but such a result will require careful analysis of how to provide meaningful benefits.

## The EBP ELSI Committee and Moving Forward

To provide EBP with advice concerning many of these ethical, legal, and social issues, EBP has convened a committee, the ELSI Committee, currently comprising 15 members with expertise in a wide range of disciplines, including anthropology, bioethics, conservation science, economics, genomics, law, public health, and science policy (45). The committee has grown since its inception and is likely to grow further in an effort to better represent the diversity of cultures, nationalities, disciplines, and fields related to EBP.

In this advisory capacity, the EBP ELSI Committee plans to draft and distribute discussion papers, guidelines, and white papers concerning the issues EBP researchers face and will face as the project gains momentum. Where appropriate, the EBP ELSI Committee will invite perspectives from the public to assist in its recommendations and to better understand the issues presented by EBP’s work. For a project such as this, we are aware of the complex issues concerning public participation, but we are nonetheless committed to inviting perspectives from a wide range of the public. The EBP ELSI Committee will also serve as a standing consultancy to researchers on EBP member projects working through specific, ad hoc issues brought to the ELSI Committee. For a project of its size and complexity, the EBP ELSI Committee recognizes that unanimity on most issues facing EBP member projects is unlikely. However, the ELSI Committee will strive for consensus, pragmatism, and equity in its recommendations.

Despite the sheer complexity of the issues described above, we believe they can largely be resolved, or, at the least, operationalized such that EBP researchers have the capacity to make fair, equitable, legal, and practical decisions about their sequencing projects. We do not mean to suggest that we have solutions—or a single, grand, overarching ELSI solution—for all sequencing projects under EBP’s umbrella. To the contrary: Many recommendations will be clear about the need for individualized and careful determinations about what to do for any given project. This means providing guidance on furthering some of the best practices and societal concerns described above, including ways of separately identifying and recognizing sample collection from Indigenous Territories, e.g., through additional metadata fields. The EBP ELSI Committee will also advocate that researchers include statements of their adherence to many of the ethical principles described above in their published work. Because these details and requirements are moving targets, we anticipate that these recommendations will be periodically revisited and updated as a core part of the EBP ELSI Committee’s work.

## Conclusion

The ethical, legal, and social issues that confront the EBP are daunting, both for their administrative complexity and for deep questions they raise about science and justice in what has long been an unfair world. However, the potential benefits for science—and, more importantly, for humanity and the entire biosphere—are great enough for us to make every effort to succeed. After all, a species intelligent enough to have the technical ability to sequence the genomes of all eukaryotic life should equally take on the responsibility to work successfully on the societal challenges that this project creates.

## Data Availability

There are no data underlying this work.
